# Does polyploidy inhibit sex chromosome evolution in angiosperms?

**DOI:** 10.3389/fpls.2022.976765

**Published:** 2022-09-23

**Authors:** Li He, Elvira Hörandl

**Affiliations:** ^1^Eastern China Conservation Centre for Wild Endangered Plant Resources, Shanghai Chenshan Botanical Garden, Shanghai, China; ^2^Department of Systematics, Biodiversity and Evolution of Plants, University of Göttingen, Göttingen, Germany

**Keywords:** angiosperms, dioecy, sex chromosomes, autopolyploidy, allopolyploidy

## Abstract

Dioecy is rare in flowering plants (5–6% of species), but is often controlled genetically by sex-linked regions (SLRs). It has so far been unclear whether, polyploidy affects sex chromosome evolution, as it does in animals, though polyploidy is quite common in angiosperms, including in dioecious species. Plants could be different, as, unlike many animal systems, degenerated sex chromosomes, are uncommon in plants. Here we consider sex determination in plants and plant-specific factors, and propose that constraints created at the origin of polyploids limit successful polyploidization of species with SLRs. We consider the most likely case of a polyploid of a dioecious diploid with an established SLR, and discuss the outcome in autopolyploids and allopolyploids. The most stable system possibly has an SLR on just one chromosome, with a strongly dominant genetic factor in the heterogametic sex (e.g., xxxY male in a tetraploid). If recombination occurs with its homolog, this will prevent Y chromosome degeneration. Polyploidy may also allow for reversibility of multiplied Z or X chromosomes into autosomes. Otherwise, low dosage of Y-linked SLRs compared to their multiple homologous x copies may cause loss of reliable sex-determination at higher ploidy levels. We discuss some questions that can be studied using genome sequencing, chromosome level-assemblies, gene expression studies and analysis of loci under selection.

## Introduction

**FIGURE 1:**
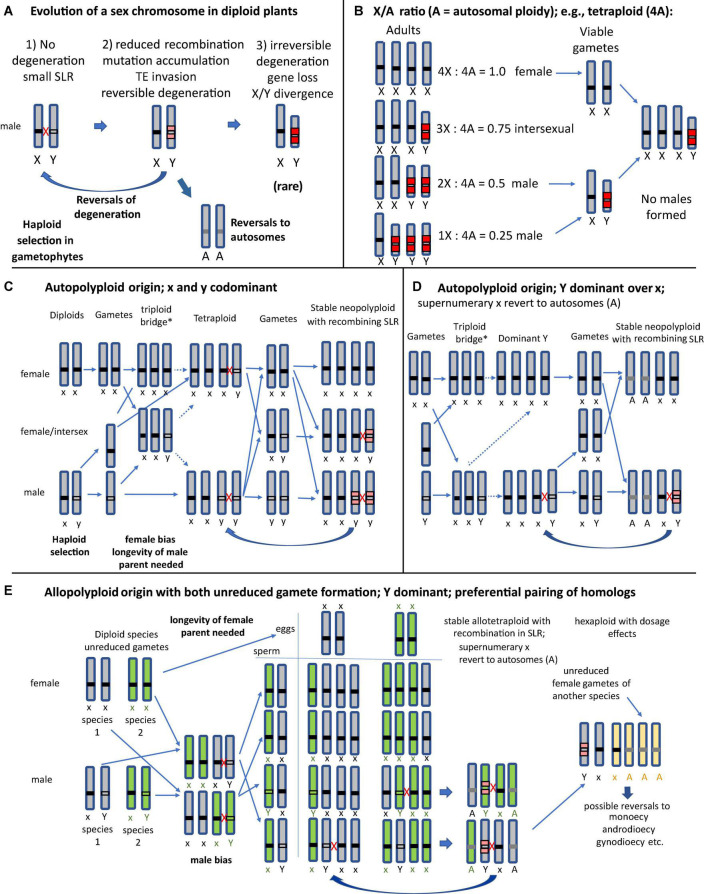
Evolutionary scenarios of SLR-bearing chromosomes and their homologs in different possible types of polyploid plants under male heterogamety, assuming different possible types of sex-determination; A, autosomes. The thick arrows indicate evolutionary changes, and the thin ones indicate crosses and gamete formation, and when the lines are dashed, this indicates that the cross has low fertility. Red crosses indicate recombination between chromosomes that pair at meiosis (for general meiosis behavior of polyploids see Supplementary Figure 1). **(A)** The three main phases of sex chromosome evolution in a diploid dioecious progenitor of a polyploid species; many plants appear to be in stages with no, or minor, degeneration. Reversals to autosomes can occur when the ancestral X-Y pair is replaces by a new set of sex-determining chromosomes. **(B)** Classical “balance” model by Muller (1925) for sex determination via the X/A ratio in species with degenerated sex chromosomes, as in many animals (including *Drosophila*, nematodes and, with female heterogamety, Lepidoptera) (Bachtrog et al., 2014), showing that polyploids can neither maintain stable X/Y ratios nor restore the heterogametic sex. This form of sex-determination appears to be rare in angiosperms. **(C)** An autopolyploid plant originated via unreduced female gametes (termed a “triploid bridge,” because a triploid generation is initially formed by unreduced gametes of one parent, and can produce haploid, diploid, and triploid gametes; the * denotes low fertility of this generation). The diagram shows the expected progeny types assuming codominant expression of a Y-linked male-determining factor, and no preferential pairing of homologs (possible xyyy genotypes not shown). **(D)** Autopolyploid with a dominant Y-linked factor; xxxY genotypes represent fertile males. After some time two of the x may revert to autosomes A, whereas recombination between the xY pair prevents degeneration. **(E)** Allopolyploid origins via unreduced gametes of both parents (shown in green and gray), and with a dominant Y-linked maleness factor. Homologs of the same parent will preferentially pair at meiosis (see also Supplementary Figure 1). Initially only xxxY male allopolyploids can be formed and produce heterozygous male gametes (see text). Backcrossing with unreduced gametes of female parents can produce various tetraploid hybrid genotypes, including both xxxx females and xxxY males, resulting in stable populations. Here also two of the x may revert to autosomes **(A)**, and only one possible recombining xY pair (derived from the same progenitor) remains. Further hybridization with a third species can result in a hexaploid, which eventually results in reversal to a non-dioecious system.

Dioecy, the sexual system with separate female and male individuals, is phylogenetically widespread in angiosperms, but occurs only in 5–6% of species (Ming et al., 2011; Renner, 2014). Functional hermaphroditism with hermaphrodite flowers is the most common system, and the transition to dioecy can involve gynodioecy, monoecy, or other sexual systems (Darwin, 1877; Richards, 1997). Dioecy in many plants is under genetic control, with a sex-linked locus or region (SLR). We term such chromosomes “SLR bearing,” but here restrict the definition of sex chromosomes (following; Charlesworth, 2015) to mean chromosomes carrying completely sex-linked regions that include many genes and do not recombine. This distinction recognizes a key aspect of such “classical” sex chromosomes, their lack of recombination, which can arise in several different ways (Charlesworth, 2022), and which allows accumulation of deleterious mutations, resulting in degenerated sex chromosomes like those in mammals and *Drosophila* (Muller, 1918; Bachtrog, 2008). One hypothesis involves selection favoring the suppression of recombination between the sex-determining locus and linked sexually antagonistic polymorphic genes (Fisher, 1931; Rice, 1987). Suppression of recombination, however, might also result from neutral sequence divergence (Jeffries et al., 2021). Lenormand and Roze (2022) recently proposed a model for stepwise evolution of sex chromosomes via “lucky inversions” that prevent recombination. Genetic sex-determination has evolved independently multiple times in plants, but many have remained in the homomorphic state, suggesting that recombination has often not become suppressed, and that their SLRs carry just a sex-determining gene (or a few physically close and closely clinked sex-determining genes), explaining why degeneration have not occurred in these species. Eventually, ancestral sex chromosomes can be replaced by a new set of sex-determining chromosomes, and revert to autosomes (Vicoso, 2019; Figure 1A).

Many angiosperms are polyploid, having more than two chromosome sets (Van De Peer et al., 2017). Polyploidy is regarded an important evolutionary mechanism for angiosperms (e.g., Comai, 2005; Soltis and Soltis, 2009, 2016; Abbott et al., 2013; Hörandl, 2022). All angiosperms share an ancient genome duplication (Jiao et al., 2011), and 25–35% of species are more recently evolved polyploids of this already polyploid state (Landis et al., 2018). Unlike most animal polyploids, most polyploid plants are viable and can reproduce sexually (e.g., Levin, 2002; Comai, 2005). Asexual reproduction (apomixis) occurs in less than 1% of species and is not necessarily correlated to polyploidy (Mogie, 1992; Hojsgaard et al., 2014; Hojsgaard and Hörandl, 2019). In animals, the reason for the rarity of polyploidization is thought to be because it disturbs the “balance” system of sex determination by the X/autosome ratio (Muller, 1925). Such systems are found in species with extremely degenerated sex chromosomes in the heterogametic sex like the *Drosophila* Y (Orr, 1990). In this case, the heterogametic sex in a tetraploid (XXYY) will only produce heterozygous (XY) gametes if X-X and Y-Y pairs form preferentially at meiosis; since YY gametes are not viable, the heterogametic sex will be lost, resulting in rapid extinction (Orr, 1990; Figure 1B). Although sex chromosomes of angiosperms have been studied for a century (reviewed by Westergaard, 1958), less attention has been given to polyploidy in dioecious plants. A correlation analysis of polyploidy and sexual systems on c. 1000 angiosperm species found that polyploidy is associated with sexual dimorphism (gynodioecy, androdioecy, and dioecy), but not specifically with dioecy, and that patterns are highly clade-specific (Glick et al., 2016). For many dioecious tropical trees and shrubs, however, chromosome numbers and ploidy levels are unknown, and more work is needed.

Knowledge about sex chromosome evolution in polyploid plants is also scanty. Modern review articles about SLRs and genetic sex determination in plants focus on diploid systems (Feng et al., 2020; Charlesworth, 2021; Renner and Müller, 2021). The recently launched database,^1^ included 166 angiosperm species with sex chromosomes or SLR-regions, of which 110 (66.3%) are classified as diploids, vs. only 20 (12%) as polyploids (Baránková et al., 2020), suggesting that polyploidy would not be highly correlated with sex chromosome evolution. However, the data are not yet clear enough, as 36 of the dioecious species (21.7%) have unknown ploidy levels (Baránková et al., 2020). Moreover, species-rich plant orders and families that experienced genome duplications before their diversification (Soltis et al., 2009), are hardly represented (only six cases in Brassicales, four in Poaceae, zero in Fabaceae, and Solanaceae), yet dioecy is present in all of them.

On the other hand, it is already known that several entirely dioecious lineages include polyploid series (Westergaard, 1958). Polyploidy may trigger shifts to dioecy (via different pathways reviewed by Ashman et al., 2013; Henry et al., 2018) speculated that whole-genome duplication could favor shifts to dioecy via evolution of sex-determining loci in duplicated genes. However, few such shifts are documented, except in *Fragaria* (Tennessen et al., 2016) where dioecy evolved in highly diploidized octoploids that arose from diploid, hermaphroditic progenitors (Tennessen et al., 2014). Most dioecious polyploids probably arose from diploid species with established SLR systems (e.g., *Salix polyclona*, He et al., 2022). Almost all angiosperms in which sex chromosome evolution is currently studied, including kiwi, papaya, persimmon, asparagus, strawberry, are cultivated plants (Henry et al., 2018), and little is known about sex chromosome evolution in natural polyploid systems. However, some examples (Table 1) include the tetraploids *Rumex acetosella* (Cunado et al., 2007) and hexaploid *Mercurialis annua* (Gerchen et al., 2022) all with male heterogamety. We briefly summarize sex chromosome evolution in diploids, and focus here on models of polyploidization from progenitors with established diploid non-degenerated systems (XY), and discuss how the presence of SLR-bearing chromosomes may affect new polyploids, and the cytological consequences, and whether polyploidy favors breakdown of dioecy, or causes reversals of X (or Z) chromosomes back to autosomes.

**TABLE 1:** Examples of polyploid plants with known sex determination systems.

Taxa	Ploidy level	Male or female heterogamety	Sex determination system	References
*Diospyros kaki*	Hexaploid	Male	XXXXXX/XXXXXY or XXXXYY	Akagi et al., 2016
*Fragaria chiloensis*	Octoploid	Female	ZW/ZZ	Tennessen et al., 2016; Cauret et al., 2022
*Melandrium album (Silene latifolia)*	Tetraploid	Male	XXXX/XXXY	Warmke and Blakeslee, 1939
*Mercurialis annua*	Hexaploid	Male	XXXXXX/XXXXXY?	Gerchen et al., 2022
*Rumex acetosella*	Tetraploid	Male	XX/XY	Cunado et al., 2007
*Salix polyclona*	Tetraploid	Female	ZZZW/ZZZZ?	He et al., 2022

## Sex chromosome evolution in diploid plants

In sessile organisms, separation of the sexes in different individuals reduces the chances of successful matings (Darwin, 1876; Hörandl and Hadacek, 2020). One factor favoring the evolution of dioecy in plants, and maintaining it over long evolutionary times, is prevention of self-fertilization and consequent inbreeding depression (Darwin, 1877; Renner, 2014). This is particularly important in wind-pollinated, long-lived plants, including tropical trees.

Dioecy and sex-linked regions evolved multiple times in angiosperms (Renner, 2014; Feng et al., 2020). Evolution from a functionally hermaphroditic ancestor requires two mutations, and both currently known plant sex-determining systems involve a gene whose action promotes one sex function and suppresses the other (review e.g., in Renner and Müller, 2021). Evolution via a turnover event in an ancestrally dioecious species will also usually involve an active maleness or femaleness factor, as the new factor must act in the presence of the ancestral one (Bull, 1983). *Rumex acetosa* and *R. thyrsiflorus* are exceptions, as sex in these species is determined by a balance system similar to that in *Drosophila* (Zuk, 1963). This balance system did not function in synthetic allopolyploid offspring (Zuk, 1963).

Charlesworth (2021) reviews three major phases of recombination and genetic degeneration (Figure 1A). Initially, SLRs are often small and do not show degeneration because there are too few sites in the completely non-recombining region, and purifying selection prevents mutation accumulation. A second phase may occur, in which recombination becomes suppressed in a larger region, allowing loss of gene functions, though the genes are still present; this weakens purifying selection on Y (or W) linked genes, allowing X-Y sequence divergence. If suppressed recombination persists for long enough in a large enough genome region, deletion of genes is expected to follow, producing highly degenerated sex chromosomes. The time needed for this stage of degeneration to be reached is unknown, and could differ between species, as it depends on mutation rates and population sizes (Bachtrog, 2008).

However, SLR regions of dioecious plants may be less subject to accumulation of deleterious mutations than in animals, because haploid gametophytes express up to 65% of genes (Joseph and Kirkpatrick, 2004; Gorelick, 2005; Cronk, 2022; Mank, 2022). Hence, haploid selection is probably much stronger in plants than in animals (Otto et al., 2015), and could prevent sex chromosome degeneration. Indeed, YY (or WW) genotypes are viable in diploid dioecious plants at first phase (Ming et al., 2011), which is consistent with purifying selection eliminating deleterious mutations in haploid Y (or W) chromosomes in gametophytes (though other alternatives, including recent origins, or small sizes of sex-linked regions, are probably also important).

Degeneration will not happen if recombination occurs. Dioecy in plants restricts sexual selection mostly to post-pollination processes (Hörandl and Hadacek, 2020). Hence, selection might favor sexually antagonistic mutations in sex-linked genes less often than in animals. Therefore factors other than sexual antagonism may be required for dioecious plants to evolve extensive fully sex-linked regions and undergo degeneration (e.g., Lenormand and Roze, 2022).

## Sex chromosomes in polyploid plants

### Cytological mechanisms

Polyploids are divided into two major types, autopolyploids that originate within one species, and those originating after hybridization between species (allopolyploids). The chromosomes of new autopolyploids may form multivalents or rings, as they are not diverged and do not pair preferentially at meiosis (e.g., Stebbins, 1947; Comai, 2005). Allopolyploids have two diverged chromosome sets (homoeologs); chromosomes derived from the same parent species show preferential pairing, resulting in more or less regular bivalent formation, whereas homoeolog pairing is less frequent (Mason and Wendel, 2020; Supplementary Figure 1). Therefore, allopolyploids maintain fixed heterozygosity for the different alleles derived from the two parental species and carried by the homoeologous chromosomes (Comai, 2005; Glover et al., 2016). These mechanisms have consequences for SLR-bearing chromosomes.

Autopolyploids originate mainly via the “triploid bridge” process (Figures 1C,D; Ramsey and Schemske, 1998). The major constraint preventing establishment of such autopolyploids is male or female bias and reduced fertility in the triploid generation and possible general instability of meiosis, resulting in aneuploid progeny and low fertility (e.g., Comai, 2005). But its unreduced (triploid) gametes can fuse during fertilization with haploid gametes from a parent of the other sex, potentially establishing a cytologically stable autotetraploid. Various tetraploid sex chromosome configurations are possible, including xxyy (small captions mean codominant or recessive versus dominant Y) genotypes, which could produce xx, xy, and yy gametophytes and gametes (Figure 1C). If Y is dominant, xxxx females and xxxY males may emerge, constituting a stable population (Figure 1D), though sex-determination in autotetraploid dioecious plants with homomorphic sex chromosomes have been little studied (Spoelhof et al., 2017). In the heterogametic sex, the SLR-bearing chromosome can pair and (if the Y-linked region is not rearranged or otherwise prevented from recombining with the x) it may continue to recombine with any of the other three x chromosomes during meiosis, preventing degeneration (Figures 1C,D).

Allopolyploidization rarely involves a triploid bridge, but mostly arises after fertilization between unreduced gametes derived from two parental species or hybrids (Ramsey and Schemske, 1998). A first factor limiting production of such populations is that both parents must have the same XY or WZ system, which can differ within a genus (e.g., *Salix* and *Silene*) (Balounova et al., 2019; Martin et al., 2019; Gulyaev et al., 2022). Second, the newly formed polyploid will be in the minority and, as single individuals of dioecious plants cannot self-fertilize, they will backcross to the parents, which creates low fitness progeny (Levin, 1975; Ramsey and Schemske, 1998). Third, reproduction of the newly formed polyploid requires fertilization by a plant of the other sex (see Figures 1C,E); thus, they must live long enough to overlap with offspring generations. They will also have to form unreduced gametes, because only fusion of unreduced male and female gametes from the two dioecious parent species will produce tetraploids with the heterogametic sex (e.g., xx_1_xY_2_ male, _1_ from species 1 and _2_ from species 2). To produce males, the Y-linked male-determining gene must be dominant, because male xx_1_yy_2_ genotypes cannot be formed via unreduced gametes of allopolyploids because the female parent can only produce xx eggs and the male parent mostly xY pollen (Figure 1E). Somatic doubling is rare in nature (Ramsey and Schemske, 1998), and hence doubling of a x_1_y_2_ male diploid interspecific hybrid together with doubling a xx female is unlikely to happen frequently enough to establish populations. The xx_1_xY_2_ allotetraploid will produce male flowers and x_1_Y_2_ and x_1_x_2_ gametes. Its SLR-bearing chromosome Y will preferentially pair with the x from the same parent, resulting in four male heterozygous gamete classes. The formation of the next hybrid generation requires crossing with an unreduced female gamete derived from the other parent; only if this occurs, a stable cohort of allotetraploids with xxxY males and xxxx females can form. Most plants can produce fertile heterogametic males with only one copy of the Y chromosomes (XXXY) and females (XXXX) (e.g., *Melandrium album* = *Silene latifolia*) (Warmke and Blakeslee, 1939; Westergaard, 1958; Henry et al., 2018). This system can potentially be maintained for higher polyploids (Westergaard, 1958). Similar considerations apply to female heterogamety.

As in autopolyploids (Figure 1E), less differentiated Y chromosomes might recombine with the conspecific X and prevent further degeneration in polyploids (Charlesworth et al., 2021). On the other hand, the presence of duplicated sex chromosomes might increase recombination rates between the SLRs of autopolyploids (Pecinka et al., 2011; Glover et al., 2016). Furthermore, polyploidization can also increase the recombination rate of X or Z chromosomes, maintaining their gene content (Wilson and Makova, 2009). However, natural selection likely reduces crossover rates of tetraploid homologs. Gates (1926) and Wright et al. (2015) suggested that the multiplied sex chromosomes (Z or X) in polyploids can gradually transform into autosomes, and this appears to have occurred in polyploid *Rumex acetosella*, which each retained have only one pair of sex chromosomes (XY for males) (Singh, 1968; Cunado et al., 2007). The dioecious octoploid *Fragaria* (e.g., *Fragaria chiloensis*) species that arose from hermaphroditic diploid progenitors also each have only one SLR on one of the homologues in each species studied (Tennessen et al., 2016; Cauret et al., 2022), while the others may have become autosomes. The “new” autosomes could still be just like the ancestral X or Z chromosome, or their genes might be lost due to diploidization.

### Genetic degeneration

Tetraploid plants will regularly form diploid gametes, in which the effects of recessive deleterious mutations in one chromosome copy will be lessened by the function provided by the unmutated chromosome; this may mask mutations from selection (e.g., Otto and Gerstein, 2008), especially in high ploidy levels (e.g., hexaploid xxxxxY). Whether genes in a Y-linked region will degenerate more slowly in a polyploid than in a diploid plant, will probably depend on several factors, including the mutations’ dominance coefficients, as well as any differences in recombination. Whether the masking effect could lead to an accumulation of deleterious recessive mutations in the pseudoautosomal region of sex chromosomein the heterozygous state needs to be investigated.

### Consequences of polyploidy for the maintenance of dioecy

Polyploids have been observed to be associated with loss of dioecy of diploid progenitors, with changes occurring to monoecy, e.g., in hexaploid *Diospyros kaki* (Akagi et al., 2016), to hermaphroditism, e.g., in tetraploid *Empetrum* (Anderberg, 1994), or to monoecy and androdioecy in tetra- to dodecaploid *Mercurialis* (Pannell et al., 2004; Gerchen et al., 2022). In willows (*Salix*), shifts to diverse sexual systems occur in polyploids (Mirski, 2014; Mirski et al., 2017). These transitions to non-dioecious systems might be favored by selection for reproductive assurance by uniparental reproduction, especially in colonization scenarios (Ashman et al., 2013). However, also gene dosage effects and differential homeolog expression could cause lability if only one active Y interacts with multiple copies of x (unless all but one have turned into autosomes). Interestingly, introgression of a new Y chromosome from distantly related species happened at the origin of hexaploid, androdioecious *Mercurialis annua* (Gerchen et al., 2022). Such reversals to non-dioecious sexual systems may be indicators that polyploidization can prevent the maintenance of sex-determining systems for long evolutionary times, and this may be a further factor contributing to the rarity of degenerated sex chromosomes in plants. However, the shifts from dioecy to non-dioecious systems in several cultivated plants, including grape vines (Badouin et al., 2020) and papaya (Ming et al., 2007), do not involve ploidy changes, and could be due to human selection for major mutations (Henry et al., 2018). Loss of active sex-determiners, such as the maleness factors discussed above, could involve Y chromosome deletions, as observed in *Silene latifolia* (Kazama et al., 2016).

## Discussion and outlook

Further work on polyploid dioecious plants with SLRs is clearly needed. Studies on neopolyploids (or synthetic polyploids) would help test whether dioecy in polyploids is rare because of constraints preventing their origination. Second, it should be tested whether auto- and allopolyploids differ in their heterogametic chromosome constitution, as predicted. Third, recombination between sex chromosome homologs, with non-recombining regions restricted in size, probably often prevents degeneration of plant sex chromosomes, alongside selection in the haploid pollen. The sizes of regions should be investigated for both XY and ZW systems, and the times when they evolved. Finally, the role of genetic or epigenetic effects in lability of sexual systems needs further study, specifically whether lability tends to be greater in higher polyploids with only one copy of the heterogametic chromosomes (XY or ZW).

Cytogenetic studies can identify heteromorphic sex chromosomes, and these will often be degenerated W or Y chromosomes, and also detectable from sex differences in genomic coverage, but in plants these appear to be the exception. Homomorphic sex chromosomes are more difficult to detect (Palmer et al., 2019; Baránková et al., 2020), sometimes not even with genome sequencing. Genome wide association studies (GWAS) can detect sex-linked regions, but will often find large partially sex-linked regions as well as completely sex-linked ones. Genetic differentiation between the sexes (F_*ST*_) can provide a finer scale for use with less differentiated SLR-bearing chromosomes (Palmer et al., 2019; He et al., 2021) and even for polyploids (Kuhl et al., 2021). The highly accurate long HiFi (high-fidelity) reads produced by PacBio, combined with Hi-C, can generate haplotype-resolved genome assemblies of polyploids (Zhang et al., 2019; Cheng et al., 2021). Genome coverage, and of Ka/Ks ratios in divergence from related species to detect weakened efficacy of purifying selection, can detect and quantify degeneration of SLR protein-coding genes (as in examples outlined above). Analyses of gene expression via RNA seq, and epigenetic mechanisms controlling expression (including cytosin-methylation and small RNAs activity) are important for understanding the expression of different sex phenotypes.

## Author contributions

Both authors listed have made a substantial, direct, and intellectual contribution to the work, and approved it for publication.

## Footnotes

1
https://www.sexchrom.csic.es/

## References

[B1] Abbott R. Albach D. Ansell S. Arntzen J. W. Baird S. J. E. Bierne N. (2013). Hybridization and speciation. *J. Evol. Biol.* 26 229–246. 10.1111/j.1420-9101.2012.02599.x 23323997

[B2] Akagi T. Henry I. Kawai T. Comai L. Tao R. (2016). Epigenetic regulation of the sex determination gene MeGI in polyploid persimmon. *Plant Cell* 28 2905–2915. 10.1105/tpc.16.00532 27956470 PMC5240738

[B3] Anderberg A. (1994). Phylogeny of the empetraceae, with special emphasis on character evolution in the genus empetrum. *Syst. Bot.* 19 35–46. 10.2307/2419710

[B4] Ashman T. L. Kwok A. Husband B. C. (2013). Revisiting the dioecy-polyploidy association: Alternate pathways and research opportunities. *Cytog. Gen. Res.* 140 241–255. 10.1159/000353306 23838528

[B5] Bachtrog D. (2008). The temporal dynamics of processes underlying Y chromosome degeneration. *Genetics* 179 1513–1525. 10.1534/genetics.107.084012 18562655 PMC2475751

[B6] Bachtrog D. Mank J. E. Peichel C. L. Kirkpatrick M. Otto S. P. Ashman T. L. (2014). Sex determination: Why so many ways of doing it? *PLoS Biol*. 12:e1001899. 10.1371/journal.pbio.1001899 24983465 PMC4077654

[B7] Badouin H. Velt A. Gindraud F. Flutre T. Dumas V. Vautrin S. (2020). The wild grape genome sequence provides insights into the transition from dioecy to hermaphroditism during grape domestication. *Gen. Biol.* 21:223. 10.1186/s13059-020-02131-y 32892750 PMC7487632

[B8] Balounova V. Gogela R. Cegan R. Cangren P. Zluvova J. Safar J. (2019). Evolution of sex determination and heterogamety changes in section otites of the genus silene. *Sci. Rep.* 9:1045. 10.1038/s41598-018-37412-x 30705300 PMC6355844

[B9] Baránková S. Pascual Díaz J. Sultana N. Alonso Lifante M. Balant M. Barros K. (2020). Sex-chrom, a database on plant sex chromosomes. *New Phytol.* 227 1594–1604. 10.1111/nph.16635 32357248

[B10] Bull J. J. (1983). *Evolution of Sex Determining Mechanisms.* Menlo Park, CA: The Benjamin/Cummings Publishing Company, Inc.

[B11] Cauret C. M. S. Mortimer S. M. E. Roberti M. C. Ashman T.-L. Liston A. (2022). Chromosome-scale assembly with a phased sex-determining region resolves features of early Z and W chromosome differentiation in a wild octoploid strawberry. *G3 (Bethesda, Md)* 12:jkac139. 10.1093/g3journal/jkac139 35666193 PMC9339316

[B12] Charlesworth D. (2015). Plant contributions to our understanding of sex chromosome evolution. *New Phytol.* 208 52–65. 10.1111/nph.13497 26053356

[B13] Charlesworth D. (2021). When and how do sex-linked regions become sex chromosomes? *Evolution* 75 569–581. 10.1111/evo.14196 33592115

[B14] Charlesworth D. (2022). Some thoughts about the words we use for thinking about sex chromosome evolution. *Philos. Trans. R. Soc. London Seri. B Biol. Sci.* 377:20210314. 10.1098/rstb.2021.0314 35306893 PMC8935297

[B15] Charlesworth D. Bergero R. Graham C. Gardner J. Keegan K. Dyer K. A. (2021). How did the guppy Y chromosome evolve? *PLoS Genet.* 17:e1009704. 10.1371/journal.pgen.1009704 34370728 PMC8376059

[B16] Cheng H. Concepcion G. Feng X. Zhang H. Li H. (2021). Haplotype-resolved de novo assembly using phased assembly graphs with hifiasm. *Nat. Methods* 18 170–175. 10.1038/s41592-020-01056-5 33526886 PMC7961889

[B17] Comai L. (2005). The advantages and disadvantages of being polyploid. *Nat. Rev. Genet.* 6 836–846. 10.1038/nrg1711 16304599

[B18] Cronk Q. (2022). Some sexual consequences of being a plant. *Philos. Trans. R. Soc. London Seri. B Biol. Sci.* 377 20210213–20210213. 10.1098/rstb.2021.0213 PMC8935308 35306890

[B19] Cunado N. Cuñado N. Navajas Pérez R. de la Herrán R. Ruiz Rejón C. Ruiz Rejón M. (2007). The evolution of sex chromosomes in the genus rumex (polygonaceae): Identification of a new species with heteromorphic sex chromosomes. *Chrom. Res.* 15 825–833. 10.1007/s10577-007-1166-6 17899410

[B20] Darwin C. (1876). *Cross and Self-Fertilization of Plants.* London: John Murray.

[B21] Darwin C. (1877). *The Different Forms of Flowers on Plants of the Same Species.* London: John Murray. 10.5962/bhl.title.110054

[B22] Feng G. Q. Sanderson B. J. Keefover-Ring K. Liu J. Q. Ma T. Yin T. M. (2020). Pathways to sex determination in plants: How many roads lead to rome? *Curr. Opin. Plant Biol.* 54 61–68. 10.1016/j.pbi.2020.01.004 32106015

[B23] Fisher R. A. (1931). The evolution of dominance. *Biol. Rev.* 6 345–368. 10.1111/j.1469-185X.1931.tb01030.x

[B24] Gates R. (1926). Polyploidy and sex chromosomes. *Nature* 117 234. 10.1038/117234a0

[B25] Gerchen J. F. Veltsos P. Pannell J. R. (2022). Recurrent allopolyploidization, Y-chromosome introgression and the evolution of sexual systems in the plant genus mercurialis. *Philos. Trans. Biol. Sci.* 377:20210224. 10.1098/rstb.2021.0224 35306889 PMC8935306

[B26] Glick L. Sabath N. Ashman T. L. Goldberg E. Mayrose I. (2016). Polyploidy and sexual system in angiosperms: Is there an association? *Am. J. Bot.* 103 1223–1235. 10.3732/ajb.1500424 27352832

[B27] Glover N. M. Redestig H. Dessimoz C. (2016). Homoeologs: What are they and how do we infer them? *Trends Plant Sci.* 21 609–621. 10.1016/j.tplants.2016.02.005 27021699 PMC4920642

[B28] Gorelick R. (2005). Theory for why dioecious plants have equal length sex chromosomes. *Am. J. Bot.* 92 979–984. 10.3732/ajb.92.6.979 21652481

[B29] Gulyaev S. Cai X.-J. Guo F.-Y. Kikuchi S. Applequist W. Zhang Z.-X. (2022). The phylogeny of salix revealed by whole genome re-sequencing suggests different sex-determination systems in major groups of the genus. *Ann. Bot.* 129 485–498. 10.1093/aob/mcac012 35134824 PMC8944726

[B30] He L. Guo F. Y. Cai X. J. Chen H. P. Lian C. L. Wang Y. (2022). Evolutionary origin and establishment of the diploid-tetraploid complex in *Salix polyclona*. *Authorea*. 10.22541/au.165769042.25880844/v1 [Epub ahead of print]. 36843569

[B31] He L. Jia K.-H. Zhang R.-G. Wang Y. Shi T.-L. Li Z.-C. (2021). Chromosome-scale assembly of the genome of salix dunnii reveals a male-heterogametic sex determination system on chromosome 7. *Mol. Ecol. Res.* 21 1966–1982. 10.1111/1755-0998.13362 33609314 PMC8359994

[B32] Henry I. M. Akagi T. Tao R. Comai L. (2018). One hundred ways to invent the sexes: Theoretical and observed paths to dioecy in plants. *Ann. Rev. Plant Biol.* 69 553–575. 10.1146/annurev-arplant-042817-040615 29719167

[B33] Hojsgaard D. Hörandl E. (2019). The rise of apomixis in natural plant populations. *Front. Plant Sci.* 10:358. 10.3389/fpls.2019.00358 31001296 PMC6454013

[B34] Hojsgaard D. Klatt S. Baier R. Carman J. G. Hörandl E. (2014). Taxonomy and biogeography of apomixis in angiosperms and associated biodiversity characteristics. *Crit. Rev. Plant Sci.* 33 414–427. 10.1080/07352689.2014.898488 27019547 PMC4786830

[B35] Hörandl E. (2022). Novel approaches for species concepts and delimitation in polyploids and hybrids. *Plants* 11:204. 10.3390/plants11020204 35050093 PMC8781807

[B36] Hörandl E. Hadacek F. (2020). Oxygen, life forms, and the evolution of sexes in multicellular eukaryotes. *Heredity* 125 1–14. 10.1038/s41437-020-0317-9 32415185 PMC7413252

[B37] Jeffries D. Gerchen J. Scharmann M. Pannell J. R. Pannell J. (2021). A neutral model for the loss of recombination on sex chromosomes. *Philos. Trans. Biol. Sci.* 376:20200096. 10.1098/rstb.2020.0096 PMC8273504 34247504

[B38] Jiao Y. Wickett N. J. Ayyampalayam S. Chanderbali A. S. Landherr L. Ralph P. E. (2011). Ancestral polyploidy in seed plants and angiosperms. *Nature* 473 97–100. 10.1038/nature09916 21478875

[B39] Joseph S. B. Kirkpatrick M. (2004). Haploid selection in animals. *Trends Ecol. Evol.* 19 592–597. 10.1016/j.tree.2004.08.004

[B40] Kazama Y. Ishii K. Aonuma W. Ikeda T. Kawamoto H. Koizumi A. (2016). A new physical mapping approach refines the sex-determining gene positions on the silene latifolia Y-chromosome. *Sci. Rep.* 6:18917. 10.1038/srep18917 26742857 PMC4705512

[B41] Kuhl H. Guiguen Y. Hoehne C. Kreuz E. Du K. Klopp C. (2021). A 180 Myr-old female-specific genome region in sturgeon reveals the oldest known vertebrate sex determining system with undifferentiated sex chromosomes. *Philos. Trans. R. Soc. B Biol. Sci.* 376:20200089. 10.1098/rstb.2020.0089 34247507 PMC8273502

[B42] Landis J. B. Soltis D. E. Li Z. Marx H. E. Barker M. S. Tank D. C. (2018). Impact of whole-genome duplication events on diversification rates in angiosperms. *Am. J. Bot.* 105 348–363. 10.1002/ajb2.1060 29719043

[B43] Lenormand T. Roze D. (2022). Y recombination arrest and degeneration in the absence of sexual dimorphism. *Science (New York, NY)* 375 663–666. 10.1126/science.abj1813 35143289

[B44] Levin D. A. (1975). Minority cytotype exclusion in local plant populations. *Taxon* 24 35–43. 10.2307/1218997

[B45] Levin D. A. (2002). *The Role of Chromosomal Change in Plant Evolution.* Oxford: Oxford University Press.

[B46] Mank J. E. (2022). Are plant and animal sex chromosomes really all that different? *Philos. Trans. R. Soc. London Seri. B Biol. Sci.* 377:20210218. 10.1098/rstb.2021.0218 PMC8935310 35306885

[B47] Martin H. Carpentier F. Gallina S. Godé C. Schmitt E. Muyle A. (2019). Evolution of young sex chromosomes in two dioecious sister plant species with distinct sex determination systems. *Gen. Biol. Evol.* 11 350–361. 10.1093/gbe/evz001 30649306 PMC6364797

[B48] Mason A. S. Wendel J. F. (2020). Homoeologous exchanges, segmental allopolyploidy, and polyploid genome evolution. *Front. Genet*. 11:1014. 10.3389/fgene.2020.01014 33005183 PMC7485112

[B49] Ming R. Bendahmane A. Renner S. S. (2011). Sex chromosomes in land plants. *Ann. Rev. Plant Biol.* 62 485–514. 10.1146/annurev-arplant-042110-103914 21526970

[B50] Ming R. Yu Q. Moore P. H. Moore P. (2007). Sex determination in papaya. *Semin Cell Dev. Biol.* 18 401–408. 10.1016/j.semcdb.2006.11.013 17353137

[B51] Mirski P. (2014). Exceptions from dioecy and sex lability in genus salix. *Dendrobiology* 71 167–171. 10.12657/denbio.071.017

[B52] Mirski P. Brzosko E. Jedrzejczyk I. Kotowicz J. Ostrowiecka B. Wroblewska A. (2017). Genetic structure of dioecious and trioecious salix myrsinifolia populations at the border of geographic range. *Tree Genet Genom.* 13:15. 10.1007/s11295-016-1096-6

[B53] Mogie M. (1992). *The Evolution of Asexual Reproduction in Plants.* London: Chapman and Hall.

[B54] Muller H. J. (1918). Genetic variability, twin hybrids and constant hybrids, in a case of balanced lethal factors. *Genetics* 3 422–499. 10.1093/genetics/3.5.422 17245914 PMC1200446

[B55] Muller H. J. (1925). Why polyploidy is rarer in animals than in plants. *Am. Natur.* 59 346–353. 10.1086/280047

[B56] Orr H. A. (1990). Why polyploidy is rarer in animals than in plants revisited. *Am. Natur.* 136 759–770. 10.1086/285130

[B57] Otto S. P. Gerstein A. C. (2008). The evolution of haploidy and diploidy. *Curr. Biol.* 18 R1121–R1124. 10.1016/j.cub.2008.09.039 19108763

[B58] Otto S. P. Scott M. F. Immler S. (2015). Evolution of haploid selection in predominantly diploid organisms. *Proc. Natl. Acad. Sci. U.S.A.* 112 15952–15957. 10.1073/pnas.1512004112 26669442 PMC4703000

[B59] Palmer D. Rogers T. Dean R. Wright A. E. Wright A. (2019). How to identify sex chromosomes and their turnover. *Mol. Ecol.* 28 4709–4724. 10.1111/mec.15245 31538682 PMC6900093

[B60] Pannell J. Obbard D. Buggs R. J. A. Buggs R. J. A. (2004). Polyploidy and the sexual system: what can we learn from mercurialis annua? *Biol. J. Linn. Soc.* 82 547–560. 10.1111/j.1095-8312.2004.00340.x

[B61] Pecinka A. Fang W. Rehmsmeier M. Levy A. A. Scheid O. M. (2011). Polyploidization increases meiotic recombination frequency in *Arabidopsis*. *BMC Biol.* 9:24. 10.1186/1741-7007-9-24 21510849 PMC3110136

[B62] Ramsey J. Schemske D. W. (1998). Pathways, mechanisms, and rates of polyploid formation in flowering plants. *Ann. Rev. Ecol. Syst.* 29 467–501. 10.1146/annurev.ecolsys.29.1.467

[B63] Renner S. Müller N. A. (2021). Plant sex chromosomes defy evolutionary models of expanding recombination suppression and genetic degeneration. *Nat. Plants* 7 392–402. 10.1038/s41477-021-00884-3 33782581

[B64] Renner S. S. (2014). The relative and absolute frequencies of angiosperm sexual systems: Dioecy, monoecy, gynodioecy, and an updated online database. *Am. J. Bot.* 101 1588–1596. 10.3732/ajb.1400196 25326608

[B65] Rice W. R. (1987). The accumulation of sexually antagonistic genes as a selective agent promoting the evolution of reduced recombination between primitive sex chromosomes. *Evolution* 41 911–914. 10.1111/j.1558-5646.1987.tb05864.x 28564364

[B66] Richards J. A. (1997). *Plant Breeding Systems.* London: Chapman and Hall. 10.1007/978-1-4899-3043-9

[B67] Singh R. B. (1968). A dioecious polyploid in rumex acetosella. *J. Heredity* 59 168–170. 10.1093/oxfordjournals.jhered.a107676 5703373

[B68] Soltis D. E. Albert V. A. Leebens-Mack J. Bell C. D. Paterson A. H. Zheng C. F. (2009). Polyploidy and angiosperm diversification. *Am. J. Bot.* 96 336–348. 10.3732/ajb.0800079 21628192

[B69] Soltis P. S. Soltis D. E. (2009). The role of hybridization in plant speciation. *Ann. Rev. Plant Biol.* 60 561–588. 10.1146/annurev.arplant.043008.092039 19575590

[B70] Soltis P. S. Soltis D. E. (2016). Ancient WGD events as drivers of key innovations in angiosperms. *Curr. Opin. Plant Biol.* 30 159–165. 10.1016/j.pbi.2016.03.015 27064530

[B71] Spoelhof J. Soltis P. Soltis D. E. Soltis D. (2017). Pure polyploidy: Closing the gaps in autopolyploid research. *J. Syst. Evol.* 55 340–352. 10.1111/jse.12253

[B72] Stebbins G. L. (1947). Types of polyploids: their classification and significance. *Adv. Genet.* 1 403–429. 10.1016/S0065-2660(08)60490-3 20259289

[B73] Tennessen J. Govindarajulu R. Ashman T.-L. Liston A. (2014). Evolutionary origins and dynamics of octoploid strawberry subgenomes revealed by dense targeted capture linkage maps. *Gen. Biol. Evol.* 6 3295–3313. 10.1093/gbe/evu261 25477420 PMC4986458

[B74] Tennessen J. Govindarajulu R. Liston A. Ashman T.-L. (2016). Homomorphic ZW chromosomes in a wild strawberry showdistinctive recombination heterogeneity but a small sex-determining region. *New Phytol.* 211 1412–1423. 10.1111/nph.13983 27102236 PMC5074332

[B75] Van De Peer Y. Mizrachi E. Marchal K. (2017). The evolutionary significance of polyploidy. *Nat. Rev. Genet.* 18 411–424. 10.1038/nrg.2017.26 28502977

[B76] Vicoso B. (2019). Molecular and evolutionary dynamics of animal sex-chromosome turnover. *Nat. Ecol. Evol.* 3 1632–1641. 10.1038/s41559-019-1050-8 31768022

[B77] Warmke H. E. Blakeslee A. F. (1939). Sex mechanism in polyploids of melandrium. *Science* 89 391–392. 10.1126/science.89.2313.391 17742784

[B78] Westergaard M. (1958). The mechanism of sex determination in dioecious flowering plants. *Adv. Genet.* 9 217–281. 10.1016/S0065-2660(08)60163-7 13520443

[B79] Wilson M. Makova K. (2009). Genomic analyses of sex chromosome evolution. *Ann. Rev. Genom. Hum. Genet.* 10 333–354. 10.1146/annurev-genom-082908-150105 19630566

[B80] Wright K. M. Arnold B. Xue K. Surinova M. O’Connell J. Bomblies K. (2015). Selection on meiosis genes in diploid and tetraploid *Arabidopsis arenosa*. *Mol. Biol. Evol.* 32 944–955. 10.1093/molbev/msu398 25543117 PMC4379401

[B81] Zhang X. Zhang S. Zhao Q. Ming R. Tang H. Tang H. (2019). Assembly of allele-aware, chromosomal-scale autopolyploid genomes based on Hi-C data. *Nat. Plants* 5 833–845. 10.1038/s41477-019-0487-8 31383970

[B82] Zuk J. (1963). An investigation on polyploidy and sex determination within the genus rumex. *Acta Soc. Bot. Policy* 32 5–67. 10.5586/asbp.1963.001

