# Supplementary material for: Does polyploidy inhibit sex chromosome evolution in angiosperms?
Source: Front Plant Sci. 2022 Sep 23;13:976765. doi: 10.3389/fpls.2022.976765 (PMC9541106; doi:10.3389/fpls.2022.976765)
Supplement: Supplementary file 1 [file Data_Sheet_1.PDF]

Supplementary Material

1.1 Supplementary Figures

Supplementary Figure S1. Typical chromosomal configurations at meiosis for autopolyploids (left) and allopolyploids (right), (both for autosomes and sex chromosomes). Transitional types (segmental allopolyploidy) not shown here, see for discussion e.g. (Comai, 2005, Cifuentes et al., 2010, Mason and Wendel, 2020).

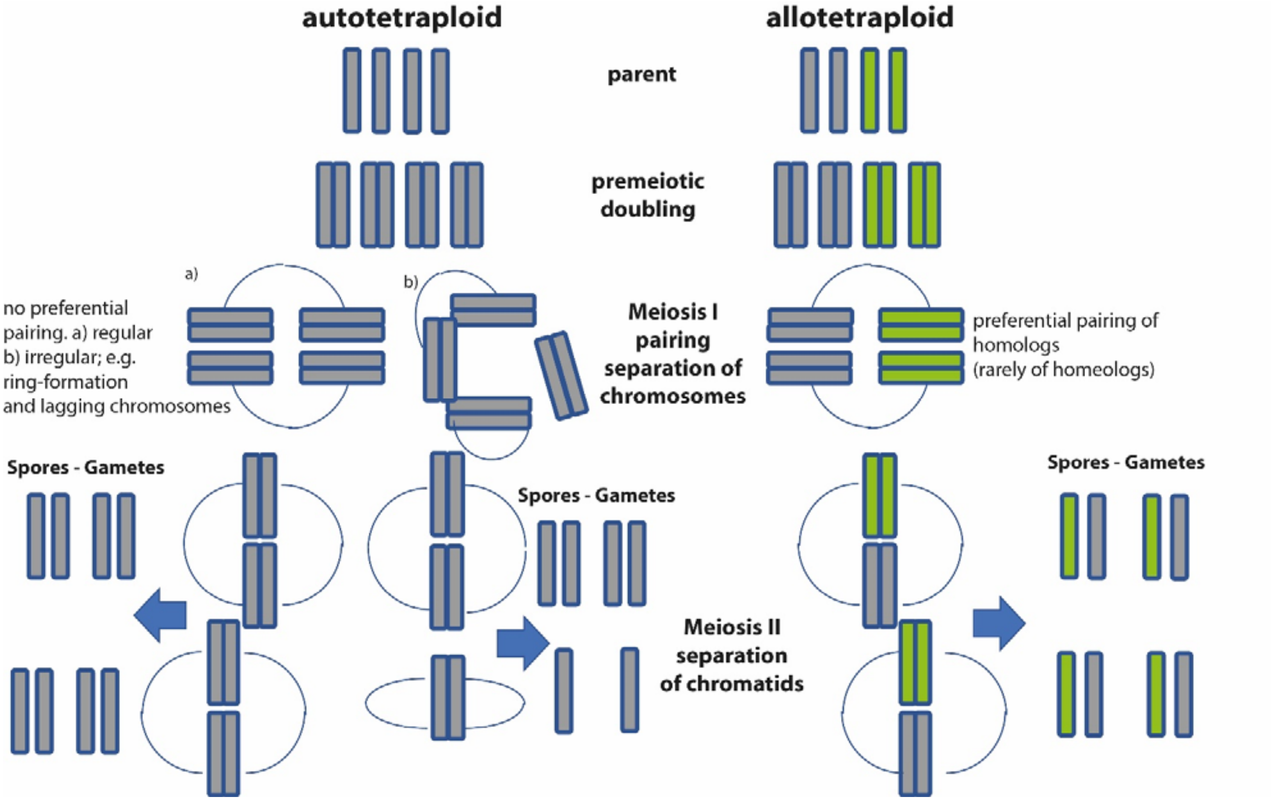

Cifuentes, M., Grandont, L., Moore, G., Chèvre, A. M., Jenczewski, E. 2010. Genetic regulation of meiosis in polyploid species: new insights into an old question. New Phytologist 186, 29-36.

Comai, L. 2005. The advantages and disadvantages of being polyploid. Nature Reviews Genetics 6, 836-846.

Mason, A. S., Wendel, J. F. 2020. Homoeologous Exchanges, Segmental Allopolyploidy, and Polyploid Genome Evolution. Front Genet 11.
